# PMR-Q&A: Development of a Bilingual Expert-Evaluated Question–Answer Dataset for Large Language Models in Physical Medicine and Rehabilitation

**DOI:** 10.3390/bioengineering13010125

**Published:** 2026-01-22

**Authors:** Muhammed Zahid Sahin, Fatma Betul Derdiyok, Serhan Ayberk Kilic, Kasim Serbest, Kemal Nas

**Affiliations:** 1 Department of Physical Medicine and Rehabilitation, Faculty of Medicine, Sakarya University Training and Research Hospital, Sakarya University, 54290 Adapazarı, Sakarya, Türkiye; zsahin@sakarya.edu.tr; 2 Department of Biomedical Engineering, Faculty of Technology, Sakarya University of Applied Sciences, 54050 Serdivan, Sakarya, Türkiye; betulderdiyok@subu.edu.tr; 3 Peakup Technology Corporation, 34400 Kağıthane, Istanbul, Türkiye; 4 Department of Mechatronics Engineering, Faculty of Technology, Sakarya University of Applied Sciences, 54050 Serdivan, Sakarya, Türkiye; kserbest@subu.edu.tr

**Keywords:** Turkish clinical NLP, bilingual parallel corpus, schema-constrained generation, LLM-based dataset generation

## Abstract

**Objectives**: This study presents the development of a bilingual, expert-evaluated question–answer (Q&A) dataset, named PMR-Q&A, designed for training large language models (LLMs) in the field of Physical Medicine and Rehabilitation (PMR). **Methods**: The dataset was created through a systematic and semi-automated framework that converts unstructured scientific texts into structured Q&A pairs. Source materials included eight core reference books, 2310 academic publications, and 323 theses covering 15 disease categories commonly encountered in PMR clinical practice. Texts were digitized using layout-aware optical character recognition (OCR), semantically segmented, and distilled through a two-pass LLM strategy employing GPT-4.1 and GPT-4.1-mini models. **Results**: The resulting dataset consists of 143,712 bilingual Q&A pairs, each annotated with metadata including disease category, reference source, and keywords. A representative subset of 3000 Q&A pairs was extracted for expert validation to evaluate the dataset’s reliability and representativeness. Statistical analyses showed that the validation sample accurately reflected the thematic and linguistic structure of the full dataset, with an average score of 1.90. **Conclusions**: The PMR-Q&A dataset is a structured and expert-evaluated resource for developing and fine-tuning domain-specific large language models, supporting research and educational efforts in the field of physical medicine and rehabilitation.

## 1. Introduction

Artificial intelligence-supported systems in the healthcare sector are becoming increasingly widespread and are being incorporated into clinical processes to a greater extent. Within clinical processes, AI-based medical imaging and information processing applications are being developed to create faster and more accurate systems for disease diagnosis, while AI-supported mobile applications and medical devices that support the rehabilitation process are continuing to be developed for disease treatment [1]. These developments, along with the continuous improvement of productive artificial intelligence systems in the field of medicine, have begun to revolutionize the field in recent years [2]. By contributing not only to clinical processes but also to medical academic literature, they are paving the way for multifaceted developments in the field of medicine [3]. In clinical terms, clinical decision support systems are presented as advanced research tools used by clinicians. With these systems, healthcare professionals can manage a more predictable process in patient diagnosis and treatment using these search tools. However, the reliability, currency, and clinical decision-making competence of these systems are among the issues that need to be considered. In this context, many academic studies have been conducted to evaluate the accuracy and reliability levels of natural language processing processes [4,5,6,7]. The vast majority of studies have shown that large language models can achieve remarkable accuracy in the medical field, and in doing so, the creation of a database from the processes involved in natural language processing has become extremely important for system reliability [8]. LLM database creation processes involve training with raw data and LLM applications, as well as question–answer datasets derived from raw data. While the system is trained directly with raw data, question patterns and answers prepared from raw data are used to develop the system’s question–answer skills. For this purpose, question–answer datasets are created from raw datasets containing literature, patient data, and other sources.

Especially in clinical decision-making processes, the source of information obtained from the database and the rationale for the interpretation provided are important for evaluating whether the accuracy of the system translates into clinical reliability [9]. In this context, many studies have been conducted on the creation of question-and-answer datasets for use in the development of AI-supported systems in the field of medicine [10,11,12]. In some studies, part of the datasets was obtained directly from raw data and quoted in the text, while in other studies, the responses to the questions were obtained from the raw data source and presented with commentary based on reasoning. Studies evaluating the applicability and reliability of these methods in the field of medicine have indicated that studies containing physician questions, answers, and comments are more reliable and have higher accuracy rates. This database stands out as one of the effective approaches to increasing reliability; however, when dealing with large datasets, the difficulty and time required to systematically collect physician comments pose a significant limitation in database creation [13].

Furthermore, the timeliness of the raw data that constitutes these databases prevents their long-term use, necessitating the retraining of the system with new, up-to-date data. In order to address the aforementioned shortcomings of LLMs, Retrieval-Augmented Generation (RAG) techniques have recently begun to be proposed. These approaches aim to address both the lack of access to current medical knowledge in models and the tendency for hallucinations to occur during training [14]. For example, the MedRAG method provided significant “zero-shot” improvements in two of the five datasets evaluated in the MIRAGE benchmark, while more modest gains were achieved in the other datasets. Nevertheless, MedRAG has demonstrated that it is an effective technique for improving the performance of medical Q&A systems by integrating external medical knowledge sources [13].

However, the creation of databases prepared for use in general medicine in specialized fields necessitates the development of LLM-supported systems specific to these fields [15]. Furthermore, since most of the existing systems are optimized only for the English language, their performance in other languages must also be evaluated independently [13,16].

To this end, this study presents an advanced PMR-Q&A (Physical Medicine and Rehabilitation Question and Answering) dataset that can be used to train LLM applications specific to the field of PMR. The dataset consists of high-quality question–answer pairs intended to support research and development efforts related to PMR-focused language models and clinical text understanding. The raw data used in creating the dataset was manually compiled and structured from basic educational resources in the field of physical medicine and rehabilitation, as well as academic publications (articles, case reports, papers, and posters) focusing on 15 different disease groups. The questions and answers generated from these sources have been carefully evaluated by physicians specializing in physical medicine and rehabilitation, with a focus on relevance and consistency for research purposes.

The PMR-Q&A dataset developed in this study is intended as a research resource for investigators working on PMR-focused language modeling and clinical text understanding. The quality and consistency of the question–answer pairs were assessed through expert evaluation, without implying clinical accuracy or regulatory approval. The dataset is designed to support methodological research on context-aware and domain-specific AI systems in PMR, rather than direct clinical deployment. In addition, the dataset may serve as a foundation for future experimental studies exploring the development and evaluation of AI-based decision support prototypes under appropriate validation and oversight frameworks.

### Related Work

When reviewing the literature on question and answering (Q&A) systems developed in the field of medicine, it is notable that datasets consisting largely of multiple-choice questions compiled from medical licensing exams are used [13,15,17] (See Table 1). While such datasets provide a suitable basis for expert-level assessments, datasets created from structured data, such as numerical clinical measurements, are still limited in number. Indeed, the BPQA dataset presented by Hang and others [18] is one of the rare examples that tests the interpretability of numerical data, such as blood pressure, by language models.

Different methodological approaches have been adopted in the creation of datasets. In some studies, questions and answers were created by extracting them directly from the text; however, these methods are mostly based on superficial information and have limited practical value because they inadequately reflect clinical reasoning. More recent studies aiming to address this limitation have shifted toward a process of inference and justification-based response generation based on scanning the source data rather than directly selecting responses to questions [20]. It is frequently emphasized in the literature that such systems produce more meaningful and explainable answers, especially when faced with complex questions, but at the same time require the verifiability of the reasons [22,23].

In this context, studies using transformer-based models are noteworthy. In particular, these models are reported to outperform traditional methods in generating long and detailed responses. For example, in the study by Wen and others [20], the explanatory quality of answers to “why” questions was improved using BERT-based models; Zhu and others [24] demonstrated that LLMs with a multi-agent architecture achieved higher accuracy in complex reasoning tasks. In addition, Goenaga and others [15] have developed more reliable and explainable systems with models that automatically extract the arguments behind the answers to questions. Such studies show that the integration of human expert opinion-based verification processes, which are considered the “gold standard,” increases the reliability of AI-based systems. However, one of the major challenges faced by medical Q&A systems is ensuring that information is up to date. In this context, the RAG approach has emerged as a hybrid method that enables LLMs to generate responses by retrieving information from external sources [14]. Findings in the literature show that RAG-based systems reduce the rate of hallucinations and increase the level of recency of responses. However, it has also been noted that these systems experience performance loss, especially in languages other than English. This situation has highlighted the need to develop multilingual datasets [8,13].

Another common issue highlighted in the literature is that LLMs still struggle with scenarios requiring complex clinical reasoning, and that developments in this area remain insufficient. In addition, uncertainties, missing explanations, or structural errors in training data can negatively affect model performance. Therefore, the development of high-quality datasets that have been validated by experts in the field is critical for the reliability of model outputs [20]. However, there is a need to create specialized, reliable datasets in medical fields [25].

In this context, when current studies and the issues in question are evaluated holistically, it is notable that while various Q&A datasets have been developed in the medical field [13,15,17], there remains a limited number of specialized studies in medical branches [21]. A review of the literature reveals a clear gap in the need for a question-and-answer (Q&A) dataset that can be used in the field of Physical Medicine and Rehabilitation (PMR). Furthermore, when evaluating existing studies, it is clear that these datasets are largely English-focused, with a limited number of examples prepared in languages other than English [26,27]. The number of studies on Turkish medical Q&A datasets is also limited [28]; moreover, no Turkish Q&A dataset developed specifically for the field of Physical Medicine and Rehabilitation (PMR) has been found in the literature.

Furthermore, while various studies evaluating the validity, reliability, and performance of medical Q&A datasets exist in the literature [18,21], no Q&A dataset prepared in Turkish and evaluated by field experts has been found in the field of PM&R. This situation indicates a significant need for the development of a high-quality Q&A dataset that is both in English–Turkish, specific to the FTR field, and includes expert evaluation.

This study aims to develop a Turkish–English dataset with an explanatory question–answer structure specific to the field of Physical Medicine and Rehabilitation (PMR), unlike general medical datasets. The limited number of Turkish medical datasets in the literature and the absence of a structured dataset specific to the PMR field constitute the main original contribution of the study. Another important factor enhancing the originality of the study is that the developed dataset has been evaluated by physicians specializing in PMR in terms of accuracy, scientific consistency, and clinical appropriateness. Thus, the dataset goes beyond being a study focused solely on data production and becomes a reliable and high-quality reference source for natural language processing (NLP)-based systems. Only scientific articles and academic theses published in Turkish, English, and other languages were used in the training process, and data reliability was supported by expert opinions. In this respect, the study fills an important gap in the literature, particularly for Turkish NLP applications and clinical decision support systems.

## 2. Materials and Methods

### 2.1. Development of the Raw Dataset

The development of the raw dataset was initiated through the acquisition of core reference materials widely used in Physical Medicine and Rehabilitation (PM&R) specialist training and clinical practice. These materials were identified and requested by PM&R specialists actively involved in the study. Access to the relevant medical textbooks was obtained via the Database Access and Statistics System (VETIS) platform of Library Documentation Department of Sakarya University, which provides authorized online access to academic resources, specifically through the ClinicalKey and MEDLINE databases. All retrieved resources were systematically organized and archived as part of the raw dataset.

To enhance the comprehensiveness of the raw dataset, secondary academic sources—including peer-reviewed journal articles, conference proceedings, and case reports—were planned to be incorporated in addition to core textbooks. Accordingly, a structured literature search was conducted across Scopus, Google Scholar, and Springer databases to identify publications from the past 20 years. The search strategy combined keywords related to 14 predefined diseases with the terms “rehabilitation,” “treatment,” and “diagnosis.” These searches were performed between March and May 2025, and all eligible publications were systematically collected, categorized, and stored in a digital folder structure.

Furthermore, to ensure the inclusion of national academic output, Turkish-language postgraduate theses were retrieved from the Council of Higher Education National Thesis Center. Using the same set of disease-specific keywords, theses published within the last 20 years and accessible between March and May 2025 were downloaded, organized, and incorporated into the raw dataset using a structured archival framework.

### 2.2. Expert Evaluation in Physical Medicine and Rehabilitation

To determine the clinical validity and applicability of the developed question–answer dataset in PMR, a structured expert evaluation process was conducted. The dataset was constructed by randomly selecting 1–2 questions from various sections of each source, yielding a representative sample comprising a total of 3000 question–answer pairs. This sample was curated to reflect the overall characteristics of the dataset and subsequently prepared for expert assessment.

The evaluation was carried out by a panel consisting of three assessors: physician with 30 years of experience in the field of PMR, a resident physician currently completing specialty training in the same field, and a physiotherapist. Each expert independently reviewed and rated the question–answer pairs based on predefined criteria. In order to enhance the objectivity of the process, both qualitative judgments and quantitative scoring metrics were employed. This dual approach allowed for a more precise assessment of the dataset’s reliability, internal consistency, and clinical usability.

To define the parameters of the evaluation scale, prior studies incorporating expert assessments in dataset validation were reviewed. Specifically, the three-level scoring systems used in MedQA [17] and MedExQA [29] (assigning scores between 0 and 1 to classify responses as incorrect/irrelevant, partially correct, or accurate with explanation) and the error typologies identified in MedXpertQA [30]—including question formatting errors, inconsistencies in expression, knowledge inaccuracies, illogical responses, and source attribution validity—were taken into consideration. Based on these frameworks, a novel Quantitative Expert Evaluation Scale for PMR Datasets was developed for the present study.

The developed scale is scored as shown in Table 2; it allows each question–answer pair to be rated at four different levels in terms of scientific accuracy, logical consistency, contextual appropriateness, and clinical reliability.

Using this scale, all 3000 question–answer pairs were independently rated by the expert panel. The resulting quantitative scores provided an evidence-based reference for assessing the dataset’s scientific reliability, clinical accuracy, and educational applicability.

### 2.3. A Framework for Automated Generation of a Specialized Medical Q&A Dataset

**Figure 1:**
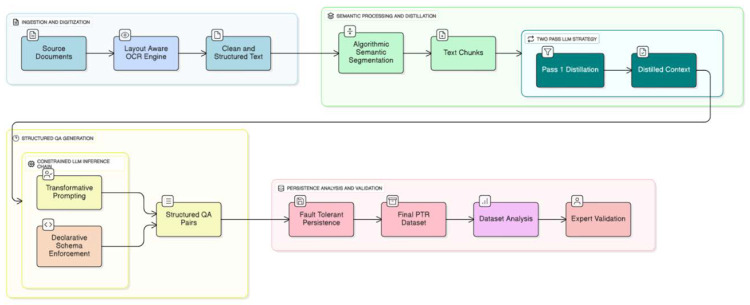
Two-stage LLM-based workflow for generating bilingual and expert-evaluated QA pairs in PMR.

The creation of the PMR dataset was accomplished through a novel, automated framework architected for the systematic conversion of unstructured scientific literature into a structured, bilingual, and high-fidelity Q&A corpus. The methodology is predicated on a multi-stage computational pipeline that integrates high-fidelity digitization and content extraction from heterogeneous source documents, followed by algorithmic semantic segmentation and contextual distillation of the extracted text, and culminates in constrained Large Language Model (LLM) inference to generate clinically relevant Q&A pairs. This approach was designed to ensure the final dataset possesses the scalability, structural consistency, and semantic depth required for fine-tuning specialized LLMs (see Figure 1).

### 2.4. Document Digitization via Layout-Aware Optical Character Recognition

The initial phase of the data pipeline necessitates the precise digitization of source documents, particularly those in complex, non-text-native formats. To address the limitations of traditional Optical Character Recognition (OCR)—which often processes text linearly and fails to capture the structural hierarchy of academic literature—we employed a deep-learning-based Visually Rich Document Understanding (VRDU) framework.

Unlike standard OCR engines that rely solely on character recognition, this methodology utilizes a multi-modal Transformer architecture (e.g., variants of LayoutLM or Document Image Transformers). This architecture integrates two distinct data streams:

1. **Visual Features:** Convolutional Neural Networks (CNNs) extract visual cues to identify document layout objects such as columns, tables, figures, and sidebars.
2. **2D Positional Embeddings:** The model maps the spatial coordinates (bounding boxes) of identified text tokens, enabling it to “understand” the relative positioning of content.

By leveraging these spatial embeddings, the system performs semantic page segmentation to classify logical blocks and reconstructs the correct reading order based on layout probability rather than simple top-down coordinates. This approach ensures that multi-column text, floating figures, and footnotes are processed in their semantic context, preventing the “logical flow corruption” typical of naive linear extraction. The output is a structured, serialization-ready text stream that preserves the semantic integrity of the original scientific manuscripts.

### 2.5. Semantic Segmentation and Contextual Distillation

To prepare the extracted text for LLM processing, a two-phase strategy involving semantic segmentation and contextual distillation was employed. For the contextual distillation stage, GPT-4.1-mini was utilized for 23% of the data, while the remaining 77% was processed using GPT-4.1, balancing efficiency and contextual accuracy.

### 2.6. Algorithmic Semantic Segmentation

The raw text extracted from each document page was partitioned into semantically coherent segments, or “chunks,” using a hierarchical, separator-based splitting algorithm [31]. This step is crucial, as LLMs operate within a finite context window (i.e., an input token limit). Simply truncating text or applying a naïve fixed-size chunking method may break sentences or cohesive thoughts, leading to loss of semantic continuity and a degradation in the quality of the generated Q&A pairs.

Our chosen algorithm prioritizes semantic integrity by attempting to split text along natural linguistic boundaries. It operates based on a predefined, ordered set of textual separators (See Algorithm 1).

Let:
**Algorithm 1:** Hierarchical Semantic Segmentation with Contextual Overlap--------------------------------------------------------------------------------Input:    T:       Raw input text    S      : Ordered separators {“\n\n”, “\n”, “.”, “;”, “,”}    C_max_:   Maximum chunk size (3000 characters)    O:       Context overlap size (300 characters)Output:    K:       Ordered list of text chunks-------------------------------------------------------------------------------- 1: function SEGMENT(block, seps) 2:     if Length(block) ≤ C_max_ then 3:         return {block}                                ▷ Base case 4:     end if 5: 6:     if seps is empty then 7:         return HARD_SPLIT(block, C_max_)             ▷ Fallback segmentation 8:     end if 9:10:     curr_sep ← seps [0]11:     parts ← Split(block, curr_sep)12:     segments ← ∅13:14:     for each p in parts do15:         segments ← segments ∪ SEGMENT(p, seps [1..])16:     return segments17: end function--------------------------------------------------------------------------------18: atomic_segs ← SEGMENT(T, S)19: K ← ∅; active_chunk ← “”20:21: for each seg in atomic_segs do22:     if Length(active_chunk) + Length(seg) ≤ C_max_ then23:         active_chunk ← active_chunk + seg24:     else25:         K ← K ∪ {active_chunk}                      ▷ Commit chunk26:         overlap ← Suffix(active_chunk, O)27:28:         ▷ Safety Check: Ensure overlap does not cause overflow29:         if Length(overlap) + Length(seg) ≤ C_max_ then30:             active_chunk ← overlap + seg31:         else32:             active_chunk ← seg                      ▷ Context sacrificed33:         end if34:     end if35: end for36:37: if active_chunk ≠ ““ then K ← K ∪ {active_chunk}38: return K---------------------------------------------------------------

Finally, the segments are merged into chunks that respect the C_max_ limit while maintaining a configured overlap O between consecutive chunks. The overlap ensures that contextual continuity is preserved at chunk boundaries, enabling the LLM to capture inter-segment relationships more effectively.

For this study, we configured a chunk size (C_max_) of 3000 characters and an overlap (O) of 300 characters. The selection of these values represents a critical balance between semantic completeness and computational constraints. A chunk size of 3000 characters is sufficiently large to encapsulate a complete clinical concept or argument, yet small enough to operate efficiently within the context window of modern LLMs, mitigating issues related to the model’s attenuated attention over very long contexts [32]. The 300-character overlap (10% of the chunk size) serves as a buffer to ensure that sentences or ideas that span across chunk boundaries are fully represented in at least one of the chunks presented to the LLM, thereby preserving contextual continuity. This heuristic-based approach is superior to fixed-size chunking, as it maximizes the probability that each chunk represents a complete and coherent unit of information, which is a critical prerequisite for high-quality, context-aware Q&A generation.

### 2.7. Contextual Distillation via a Two-Pass LLM Strategy

To enhance the quality and relevance of the generated Q&A pairs, a novel two-pass “distill-then-generate” strategy was implemented. Grounded in the principle of task decomposition [32,33], this approach enhances LLM reasoning capabilities by breaking the complex generation problem into two distinct, sequential steps. The process is formalized as follows:

1. First Pass—Contextual Distillation:

In the initial phase, a text chunk $C_{i}$ (where *i* denotes the chunk index) is processed by the LLM using a distillation prompt, $P_{distill}$. This prompt instructs the model to act as a semantic filter, performing an extractive summarization task. The LLM is directed to identify and retain only the core clinical and scientific facts, while excluding methodological details, author citations, and other non-clinical “distractor” elements.

The output of this stage is a condensed, factually focused representation of the original chunk, denoted as $C_{i}^{\prime }$ defined as:

$$C_{i}^{\prime }=LLM(P_{distill},C_{i})$$

2.  Second Pass—Q&A Generation:

The distilled context *Cᵢ*′ is then passed into a second LLM call using a generation prompt, Pgenerate. This stage performs a transformative reasoning operation: instead of summarizing, the model generates application-oriented question–answer (Q&A) pairs grounded in the distilled content. The process can be expressed as:

$$QAi=LLM(Pgenerate,{C_{i}}^{\prime })$$

This two-pass methodology provides two significant, empirically supported advantages.

### 2.8. Mitigation of Contextual Noise and Improved Signal-to-Noise Ratio

Academic literature is information-dense but often contains text (e.g., detailed statistical methods, extensive citations) that is irrelevant for generating clinical Q&A. Prior studies have shown that long and noisy contexts can negatively affect LLM performance when relevant information is embedded within them [31]. Informed by this literature, the distillation step in our pipeline is designed to reduce irrelevant content and emphasize clinically relevant text, with the goal of providing more focused input to the generation prompt.

### 2.9. Enhanced Reasoning Through Task Decomposition

Complex cognitive tasks are better handled by LLMs when broken into simpler, sequential steps, a principle demonstrated by Chain-of-Thought prompting [34]. Our two-pass strategy is an application of this principle. The first pass represents a simpler reasoning task (fact extraction and summarization), which simplifies the problem space. The second pass can then build upon this structured, distilled information to perform the more complex, abstractive reasoning required for transformative Q&A generation. This compositional approach has been shown to improve the fine-tuning and performance of LLMs on complex tasks [35].

### 2.10. Question Standardization and Transformative QA Filtering

During the second pass of Q&A generation, the raw LLM output occasionally produced questions that directly requested study-specific information (e.g., “According to this study, what was the time until full return to work for all patients?”). Such questions primarily assess information recall rather than clinical reasoning, which conflicts with the educational objectives of the PMR dataset (See Table 3).

An initial analysis of the first 5000 generated QA pairs revealed that approximately 12% of questions fell into this category. Typical examples included:

• “What was the exact improvement in WOMAC scores in this study?”

These questions are problematic because they test factual memorization instead of evaluating a clinician’s ability to apply knowledge to real-world patient scenarios. To address this issue, an algorithmic enrichment and transformation step was implemented. This procedure extracts the core clinical facts from the distilled text and reformulates them into scenario-based, problem-solving questions emphasizing practical application. For example:

• “A 45-year-old patient presents with acute low back pain of 3 days’ duration. How should symptom duration and pain characteristics guide the clinical assessment and initial management strategy?”

Following the implementation of this transformation step, a post-analysis indicated that over 95% of newly generated questions adhered to the application-oriented format, effectively eliminating study-specific recall questions and ensuring a consistently high-quality, clinically relevant dataset.

This standardization step is therefore an important component of the Q&A generation pipeline, as it is designed to encourage structured and concept-oriented responses rather than surface-level pattern matching, aligning with the dataset’s intended role as a research resource for developing and evaluating PMR-focused language models.

#### 2.10.1. Structured Q&A Generation via Constrained LLM Inference

The core of data generation relies on a carefully engineered LLM chain that combines advanced prompting with schema enforcement to produce structured, reliable output.

#### 2.10.2. Transformative Prompt Engineering and In-Context Learning

The prompt template was designed based on principles of instruction tuning [33] and in-context learning [34]. The primary directive, termed “The Golden Rule,” instructs the LLM to adopt the persona of a senior clinician creating board-exam-style questions. This re-frame the cognitive task from simple extractive Q&A (e.g., “What did the study find?”) to a more complex, transformative, reasoning-based Q&A (e.g., “What does this finding imply for a specific patient case?”). This instruction-based approach conditions the model to generate outputs that test the application and synthesis of knowledge, rather than mere information recall.

To further guide this behavior, the prompt leverages in-context learning by providing several few-shot examples. These exemplars are not merely samples of the desired output; they serve as demonstrations of the intended reasoning process. Each example explicitly contrasts a “bad” extractive question with an “excellent” transformative question derived from the same source sentence. This technique relies on the LLM’s ability to recognize patterns from a small number of demonstrations provided directly within the context window [34]. By observing these transformations, the model learns the desired input–output mapping, conditioning its generative probability distribution to favor responses that align with the demonstrated style of clinical reasoning. This ensures the generated questions possess a high degree of pedagogical value, making the resulting dataset particularly suitable for fine-tuning models intended for clinical education and decision support.

#### 2.10.3. Constrained Generation via Declarative Schema Enforcement

To guarantee the structural integrity and consistency of the generated data, a constrained generation methodology was implemented. This approach shifts validation from a post-processing step to an integral part of the generation process itself.

• Declarative Schema Definition: A strict data schema was formally defined using typed data-modeling constructs. This schema specifies the data type (e.g., string, enumerated types), descriptive metadata, and logical constraints for every field within a Q&A pair (e.g., list length constraints such as 1 ≤ n ≤ 5 for keywords). This declarative approach establishes a formal “contract” for the LLM’s output, specifying the required structure a priori.
• Automated Structured Parsing: An automated parsing layer was integrated into the generation pipeline. This layer introspects the defined data schema to dynamically generate formatting instructions. These instructions, typically in a structured format like JSON Schema, are then programmatically injected into the LLM prompt. This serves to guide the model’s generation process, constraining its output to a valid JSON object that conforms precisely to the predefined schema.

This schema enforcement strategy is a critical technical contribution, as it circumvents the need for fragile, post hoc parsing of unstructured text output (e.g., via regular expressions), a common source of errors and inconsistencies in data generation pipelines. It ensures that every generated data point is immediately machine-parseable, type-safe, and adheres to the predefined structure, guaranteeing dataset quality and consistency at scale.

#### 2.10.4. The Generation Chain as a Computational Graph

The entire generation process for a given context chunk is conceptualized as a compositional dataflow, or a directed acyclic graph, where each node represents a distinct computational step. This can be formalized as follows:

Let *C_i_*′ be the distilled context for chunk *i*, and let S be the predefined output schema. The generation function *F* yields a structured Q&A object *QAi*:

*QAi* = *F*(*C_i_*′) = (*Ψ*
**∘**
*L*
**∘***Π*)(*C_i_*′)

where

• *Π* is the Prompt Templating Function, which instantiates the transformative prompt by populating it with the contextual data *C_i_*′ and the formatting instructions derived from schema S.
• *L* is the core LLM Inference Function, which processes the instantiated prompt to generate a raw token sequence, Trawi, corresponding to the desired structured output.
• *Ψ* is the Parsing and Validation Function, which deserializes the raw text output Trawi and validates its structure and content against the schema S.

This modular, chained architecture ensures a deterministic and verifiable flow of data. Each component has a single responsibility, and the successful execution of the final step, *Ψ*, guarantees that the resulting data object, *QAi*, is structurally valid and conformant to the established schema before it is persisted.

#### 2.10.5. Data Persistence and Fault-Tolerant Workflow

The main execution script is designed for robustness during long-running data generation tasks. An incremental saving and resume-support mechanism was implemented. After each source document is fully processed, the newly generated Q&A pairs are immediately appended to the master Excel file. Before starting a new session, the script first loads the existing data and compiles a set of already processed filenames. The file discovery process then filters out these files, ensuring that the workflow only operates on new or unprocessed documents.

This approach provides critical fault tolerance. In the event of an interruption (e.g., API timeout, network failure, user cancellation), all previously completed work is preserved. The process can be restarted at any time, and it will automatically resume from the last unprocessed file, ensuring efficiency and preventing data loss.

## 3. Results

The application of the methodology described resulted in a comprehensive Q&A dataset comprising over 143,712 bilingual pairs. We uploaded the PMR-Q&A dataset and code content to the Hugging Face profile [36]. A subsequent analysis was performed to characterize the composition and thematic distribution of the dataset, providing insight into its scope and suitability for training specialized PMR language models (See Table 4).

### 3.1. Distribution of Clinical Topics

**Figure 2:**
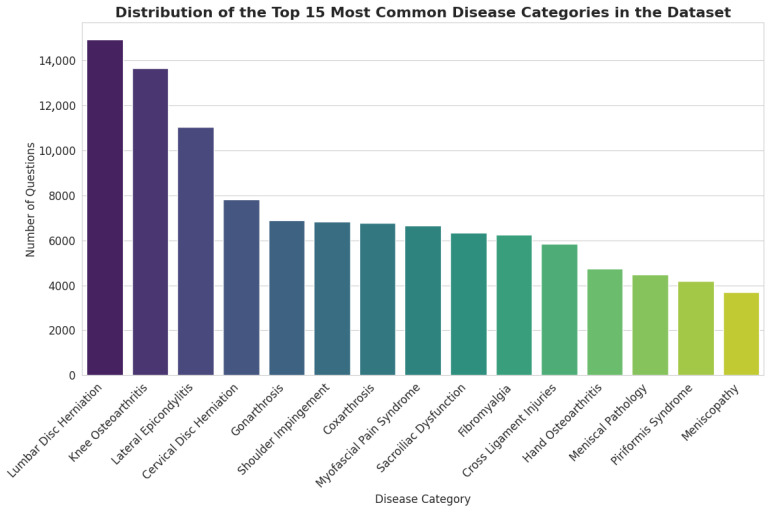
Distribution of the Top 15 Most Common Disease Categories in the Dataset.

The dataset covers a wide range of clinical conditions pertinent to PMR. As illustrated in Figure 2, the distribution of topics follows a long-tail pattern, with a concentration on common musculoskeletal disorders. The most prevalent categories include Lumbar Disc Herniation (14,927 questions), Knee Osteoarthritis (13,659 questions), and Lateral Epicondylitis (11,033 questions). This distribution reflects the high incidence of these conditions in clinical practice, ensuring that a fine-tuned model would be well-versed in the most frequently encountered patient presentations.

### 3.2. Thematic Focus via Keyword Analysis

**Figure 3:**
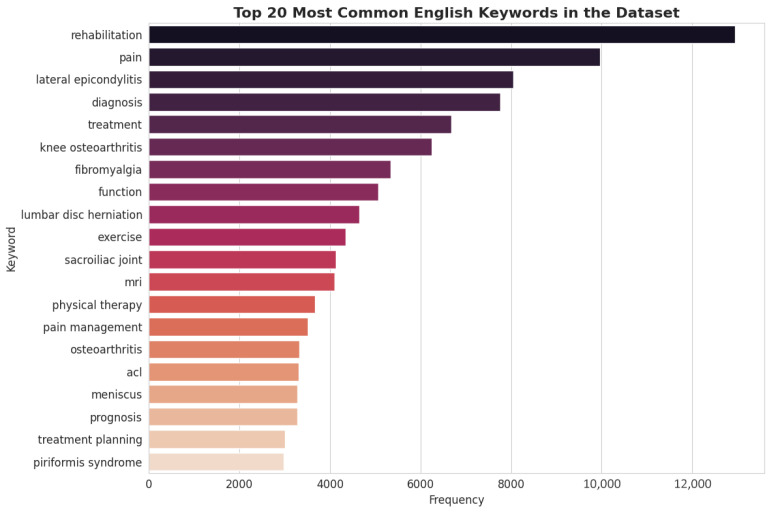
Top 20 Most English Keywords in the Dataset.

To analyze the conceptual focus of the dataset, a frequency analysis of the generated English keywords was conducted. The results, depicted in Figure 3, highlight the core themes embedded within the Q&A pairs. Terms such as “rehabilitation,” “pain,” “diagnosis,” “treatment,” and “exercise” are among the most frequent, confirming that the dataset is thematically centered on the complete clinical workflow of PMR. Furthermore, the high frequency of specific condition-related keywords like “lateral epicondylitis” and “knee osteoarthritis” aligns with disease category distribution. The presence of keywords related to diagnostic modalities (“mri”) and functional outcomes (“prognosis,” “function”) indicates a well-rounded dataset that covers not only therapeutic interventions but also the crucial aspects of assessment and patient management.

### 3.3. Analysis of Question-And-Answer Lengths

**Figure 4:**
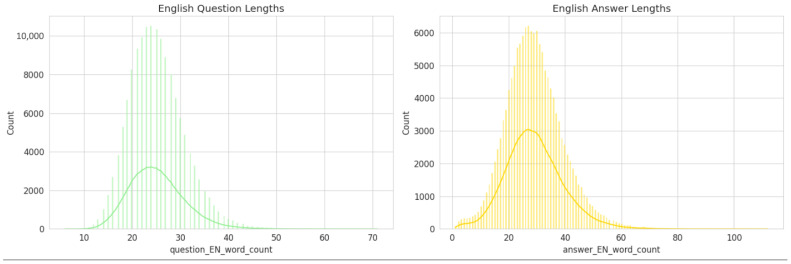
Distribution of English question-and-answer lengths in the PMR-Q&A dataset.

To better understand the linguistic characteristics of the generated dataset, the word-length distributions of English questions and answers were analyzed (See Figure 4).

The English Question Length distribution (left panel) demonstrates a near-normal pattern centered around 22–25 words per question, indicating that most generated questions are concise yet contextually informative. The narrow spread of the curve suggests a consistent formulation style, which aligns with the structured, clinically oriented nature of the dataset.

Conversely, the English Answer Length distribution (right panel) exhibits a slightly broader and right-skewed pattern, with a central tendency around 30–35 words per answer. This reflects the fact that answers typically require more elaboration, often incorporating clinical reasoning, therapeutic implications, or outcome-based statements.

Overall, this analysis indicates that the dataset maintains a balanced linguistic structure—questions remain focused and well-defined, while answers provide sufficient explanatory depth. Such consistency is essential for effective downstream training of question–answer and reasoning tasks within medical and rehabilitation-focused LLMs.

### 3.4. Analysis of the Expert Validation Sub-Dataset

To facilitate a manageable and efficient expert validation process, a representative sub-dataset of 3000 unique question–answer pairs was sampled from the full corpus of 143,712 entries. A critical step before submitting this sample for review is to verify its representativeness of the entire dataset. This section provides a comparative analysis to demonstrate that this smaller sample is a valid microcosm of the full data, ensuring that expert feedback is both relevant and scalable. The analysis compares the 3000-item sample to the complete dataset across three key dimensions: the distribution of clinical topics, the thematic focus identified through keyword frequency, and the fundamental linguistic characteristics of the questions and answers.

### 3.5. Distribution of Clinical Topics

The distribution of disease categories within the 3000-item sample closely mirrors that of the full dataset, confirming that the sample accurately reflects the clinical scope of the entire corpus. In both datasets, the most prevalent categories are Lumbar Disc Herniation, Knee Osteoarthritis, and Lateral Epicondylitis, maintaining their top rankings and relative proportions. This consistency helps focus the evaluation process on the most frequently represented clinical conditions within the dataset, reducing the risk of feedback being dominated by unrepresentative samples.

### 3.6. Thematic Focus via Keyword Analysis

A comparative analysis of keyword frequencies further reinforces the thematic alignment between the sample and the full dataset. Core concepts central to the PMR domain—such as “rehabilitation,” “pain,” “diagnosis,” and “treatment”—remain the most frequent keywords in both datasets. This alignment demonstrates that the conceptual and thematic focus of the Q&A pairs is preserved in the smaller sample. Consequently, expert review of this sample will provide valid insights into the quality and relevance of content across the primary themes of the entire corpus.

### 3.7. Linguistic Characteristics

The linguistic structure of the generated Q&A pairs is also consistently represented in the validation sample. The statistical distributions of question-and-answer lengths in the 3000-item sample are nearly identical to those of the full dataset.

• Question Length: The distribution of English question lengths in the sample maintains a near-normal pattern centered around 22–25 words.
• Answer Length: Similarly, the English answer lengths exhibit the same slightly right-skewed distribution, with a central tendency around 30–35 words.

This structural consistency indicates that the sample retains the same level of conciseness in its questions and the same degree of explanatory depth in its answers as the full dataset. This ensures that experts will be evaluating content with the same stylistic and structural properties intended for the final model.

### 3.8. Conclusion on Sample Representativeness

**Figure 5:**
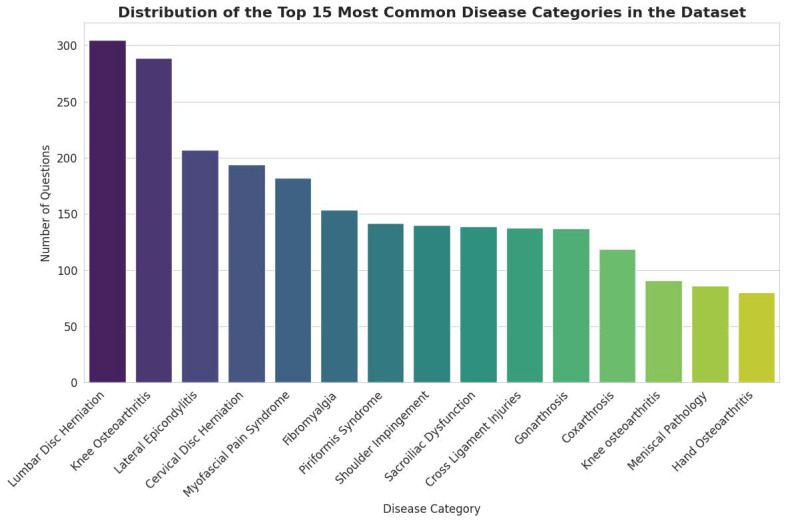
Distribution of the Top 15 Most Common Disease Categories in the Sub-dataset.

**Figure 6:**
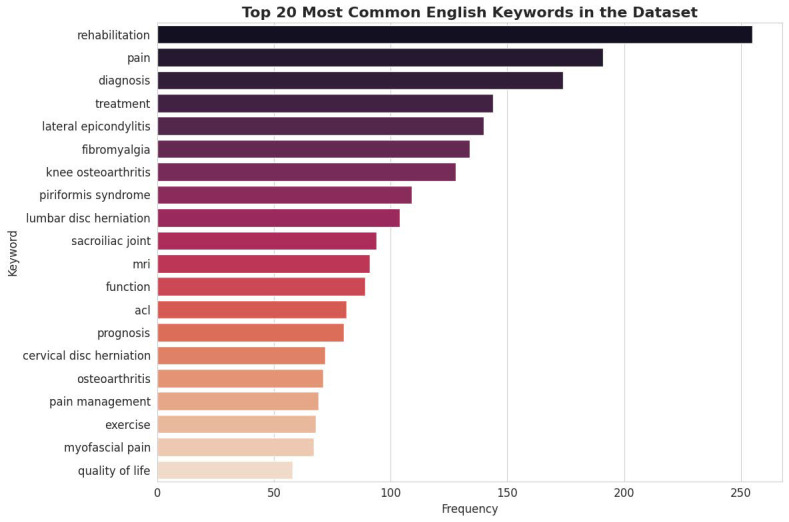
Top 20 Most Common English Keywords in the Sub-dataset.

**Figure 7:**
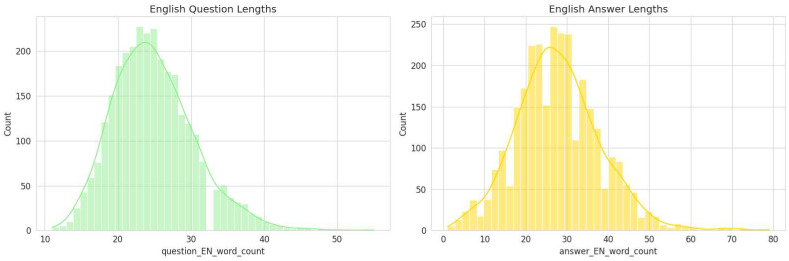
Length Distribution of English Questions and Answers in The Sub-Dataset.

In summary, the comparative analysis demonstrates a high degree of fidelity between the 3000-item validation sub-dataset and the complete 143,712-item corpus. The sample accurately preserves the distribution of clinical topics, the primary thematic focus, and the core linguistic characteristics. This congruence validates the use of the sub-dataset for expert review, ensuring that their qualitative assessments will be representative of the dataset (See Figure 5, Figure 6 and Figure 7). Although Figure 5, Figure 6 and Figure 7 visually mirror the distributions of the full dataset (Figure 2, Figure 3 and Figure 4), this similarity is intentional and serves as statistical confirmation. It visually validates that the stratified sampling method successfully preserved the long-tail distribution of disease categories and the linguistic characteristics of the corpus, proving that the validation subset is an unbiased microcosm of the full dataset.

### 3.9. Computational Infrastructure and Model Configuration:

All dataset generation and preprocessing tasks were orchestrated on a dedicated computational node equipped with 4 virtual CPUs and 16 GB of RAM. This hardware configuration was optimized to handle high-throughput API requests and large-scale JSON serialization operations efficiently. The entire data generation process was carried out continuously over a period of 23 days, ensuring comprehensive coverage of the source material.

The core semantic processing was driven by a strategic hybrid model approach utilizing both GPT-4.1 and GPT-4.1-mini. To balance computational efficiency with semantic fidelity, GPT-4.1-mini was utilized for the initial 23% of the dataset during the pilot phase, while the remaining 77% was processed using the more capable GPT-4.1 model. Although prompt engineering was rigorously optimized for both models, early analysis revealed that the smaller model (GPT-4.1-mini) exhibited limitations in handling the complex “distill-then-generate” cognitive load, demonstrating a tendency towards semantic compression loss and lower adherence to strict schema constraints.

Consequently, the pipeline was transitioned to the GPT-4.1 model for the majority of the corpus. Crucially, the outputs generated by the mini model during the pilot phase were subjected to an additional rigorous filtering and validation pass to ensure they met the high-quality standards of the main corpus before inclusion.

To ensure reproducibility and mitigate the probabilistic nature of Large Language Models across both engines, the decoding hyperparameters were strictly controlled:

• Temperature (T = 0.3): Set to a low value to prioritize determinism and factual adherence over creativity, significantly reducing the risk of hallucination.
• Top-P (*p* = 0.9): Employed nucleus sampling to maintain linguistic naturalness while truncating the tail of low-probability tokens.
• Frequency Penalty (0.0): Kept at neutral to ensure the model did not artificially avoid repeating necessary medical terminology.

This configuration established a controlled inference environment, ensuring that the generated Q&A pairs remained clinically grounded regardless of the underlying model.

### 3.10. Bilingual Alignment and Consistency

To quantify Turkish–English pairing consistency in the Q&A dataset, we computed cosine similarity between language-agnostic character n-gram TF-IDF (term frequency–inverse document frequency) vector representations of each Turkish–English Q&A pair. The mean cosine similarity was 0.168 ± 0.110 (median: 0.145). In addition, length-based consistency showed strong cross-lingual alignment: word-count correlations were r = 0.916 for Q&A pairs, r = 0.848 for questions, and r = 0.933 for answers (all *p* < 0.001). English Q&A texts were on average 12.5% longer than their Turkish counterparts (median ratio: 1.12; 5th–95th percentile: 0.94–1.33). Only 5% of pairs fell into the lowest similarity tail (≤0.034), indicating generally stable bilingual pairing with a small set of potential outliers.

### 3.11. Dataset Balance and Complexity Indicators

Across the target PMR conditions, the category distribution showed moderate imbalance (max/min ratio: 8.45), while normalized entropy remained high (0.947), suggesting broad coverage. Mean Q&A length ranged from 40.7 to 53.4 words in Turkish and from 49.1 to 58.6 words in English across categories. Although length differences were statistically significant (Kruskal–Wallis, *p* < 0.001), effect sizes were small-to-moderate (epsilon^2^ ≈ 0.04–0.10), indicating no extreme length/complexity skew.

### 3.12. Computational Resources

All dataset generation and preprocessing tasks were executed on Microsoft Azure using a standard D-series virtual machine. The VM was configured with 4 virtual CPUs, 16 GB of RAM, and a 128 GB SSD, providing sufficient computational power and memory to efficiently handle large-scale data operations. The entire data generation process was carried out continuously over a period of 23 days, ensuring comprehensive coverage and quality. The Azure environment ensured reliable performance, high I/O throughput, and scalability, allowing parallel processing of multiple data streams. This setup facilitated smooth and efficient workflow, minimizing processing bottlenecks and resulting in a high-quality dataset suitable for subsequent model training and evaluation.

### 3.13. Expert Evaluation Process of the Question–Answer Dataset

To ensure the validity and reliability of the question–answer dataset, an expert evaluation process was conducted involving three professionals. The expert panel consisted of a physician with 30 years of experience in the field of PMR, a resident physician currently completing specialty training in the same field, and a physiotherapist with extensive clinical experience.

To ensure statistical representativeness, a stratified random sampling algorithm was employed rather than simple random selection. The dataset was stratified by disease category (15 strata). The sample size for each stratum was calculated proportionally to its weight in the full corpus (e.g., since ‘Lumbar Disc Herniation’ constitutes 10.4% of the total dataset, 312 questions were sampled from this category). The sampling process was executed using a fixed random seed (random_state = 42) to ensure reproducibility.

The experts convened in a series of in-person meetings to evaluate the resulting sample of 3000 question–answer pairs. Each pair was assessed based on multiple criteria, including the accuracy of the response, logical consistency, level of detail, and clinical relevance. Specific attention was paid to ‘Citation-Grounding’ and ‘Hallucination Detection’ during the review. Experts were explicitly instructed to verify that each generated answer was factually supported by the source text. Any answer containing information not present in the source chunk or contradicting established medical guidelines was flagged as a ‘critical error’ (Score 0), acting as a manual factuality stress test.

Question–answer pairs containing correct responses but exhibiting logical inconsistencies or insufficient coherence between the question and the answer were assigned a score of 0. The number of such items was 38, representing approximately 1% of the entire dataset.

Question–answer pairs with correct answers but lacking sufficient detail and/or directly referencing the source were scored 1 point and constitute 9% of the dataset, comprising 307 questions. The first 300 items of the dataset primarily consisted of pilot samples derived from literature sources. Consequently, a relatively higher proportion of 0- and 1-point items was observed within this subset; however, their overall proportion within the complete dataset remained minimal (%10).

Following the initial 300 items, the subsequent portion of the dataset was refined through collaborative expert review. During this process, prompt adjustments were made to improve clarity, coherence, and clinical applicability. As a result, the dataset was enhanced to a level suitable for use in clinical and educational contexts. Question–answer pairs deemed accurate, relevant, and sufficiently informative were assigned a score of 2. This category comprised 2558 items, representing approximately 76% of the dataset. This finding indicates that the majority of the dataset consists of accurate and appropriately detailed question–answer pairs. It is important to note that a score of 2 represents the ‘gold standard’ for clinical safety and correctness, meaning the model output is directly usable in practice.

Additionally, question–answer pairs with higher levels of expert-assessed complexity and domain-specific detail were assigned a score of 3. A total of 97 items, corresponding to 2% of the dataset, were categorized in this group. For the English subset, 201 items, corresponding to approximately 6.7% of the dataset, were assigned the same score. The proportion of higher-scored items relative to lower-scored or insufficiently detailed entries indicates overall consistency in expert evaluation, without implying clinical validity. However, the fact that there are fewer examples scoring 2 points indicates that this content is suitable for specialized applications requiring advanced expertise, while content scoring 2 points is more highly integrated for general users and clinical application systems.

For the Turkish question–answer subset, the statistical evaluation yielded a mean score of 1.90 with a standard deviation of 0.41. Inter-rater reliability among the three PMR specialists was assessed using a two-way random-effects intraclass correlation coefficient with average measures (ICC(2,k)) [37], resulting in an ICC(2,k) value of 0.88 with a 95% confidence interval (CI) ranging from 0.85 to 0.91, indicating strong agreement among the expert evaluators. For the English question–answer subset, expert evaluation produced a comparable mean score of 2.00 with a standard deviation of 0.44, and inter-rater reliability remained similarly high (ICC(2,k) = 0.87, 95% CI: 0.84–0.90). Taken together, these results suggest consistent expert evaluation across both language subsets and a homogeneous distribution of scores at the dataset level.

Furthermore, we report per-condition descriptive statistics for the Top-15 disease categories to characterize variation across clinical topics (Table 5). According to Table 5, the mean scores of most Top-15 disease categories are consistent with the overall mean score (≈1.90). This indicates that the expert evaluation is generally consistent across different clinical topics. However, the Lateral Epicondylitis category has a lower mean score (1.396). This is mainly thought to be due to a higher proportion of question–answer pairs in this category being scored as 0–1 by the experts. Overall, the fact that scores remain within a similar range except for a single category supports that the dataset maintains a similar level of quality across different disease topics.

## 4. Discussion

This study aimed to develop an expert-evaluated Q&A dataset grounded in PMR domain knowledge, intended to support research on PMR-focused language models. The dataset was constructed using scientific research materials—including peer-reviewed articles, theses, books, and conference proceedings—focused on the most prevalent disorders encountered in the field. A total of 2641 primary sources were systematically processed, resulting in a corpus of 143,712 Q&A pairs. Throughout the process, the dataset was continuously reviewed and refined by domain experts in PMR to ensure clinical relevance, linguistic accuracy, and conceptual integrity.

Unlike many existing medical question–answer datasets based on extracting facts directly from scientific articles (prepared using medical specialty exam questions and clinical guidelines [38]), the dataset presented in this study is designed to simulate real-world clinical reasoning. The generated questions extend beyond factual recall to capture complex, scenario-based decision-making processes, reflecting situations a clinician may encounter in everyday practice. Existing medical Q&A corpora such as MedMCQA [19], emrQA [39], and MLEC-QA [40] are generally structured to cover the entirety of medical knowledge. Consequently, their question formats tend to emphasize exam-oriented recall rather than applied clinical judgment. In contrast to this situation, Cosentino et al. [41] planned to create a dataset reflecting real clinical scenarios using data obtained from raw sources supplied in the general medical field. Even Raghavan et al. [42] created a question–answer dataset using direct patient clinical data. However, as noted in a similar study by Lyu et al. [43], such datasets lack the domain-specific depth required for training reasoning-intensive models in medical subfields. Addressing this gap, our work introduces the first PMR-specific dataset built to model diagnostic, therapeutic, and decision-support reasoning within the rehabilitation domain.

One of the distinctive features of the dataset is its focus on high-prevalence clinical conditions commonly encountered in PMR. This targeted scope is intended to reflect representative clinical contexts within the field, supporting educational use and research on PMR-oriented language models.

A review of the literature reveals that several prior studies have introduced mechanisms to ensure dataset validity and clinical reliability (See Table 6). For instance, Goenaga et al. (2024) [15] employed a gold-standard evaluation framework combining expert assessments to verify the correctness of generated answers. Similarly, Kell et al. [38] and Zuo et al. [30] integrated expert validation stages to quantitatively assess clinical soundness. Kim et al. [29], in their work on MedExQA, utilized a three-expert rating system classifying responses as biased, partially correct, or fully correct. Inspired by these approaches, our study incorporated expert validation by two board-certified physiatrists and one physiotherapist, extending the evaluation framework with an additional category—advanced-level expertise required—to capture academic depth beyond standard clinical correctness. The validation subset comprised 3000 representative Q&A pairs, proportionally sampled across all data sources. This extensive validation design exceeds the scope of comparable studies such as Kim et al. [29], who evaluated only 25 instances, thereby improving generalizability and robustness of the results.

Building on the expert-based evaluation of the medical question–answer dataset, ethical safeguards and structural safety mechanisms represent a complementary layer of trustworthiness beyond clinical review alone. While physician-led assessment ensures medical plausibility, relevance, and alignment with current clinical practice, additional constraints are necessary to govern how such datasets may shape the behavior of generative AI systems trained on them.

In this study, schema-based enforcement was employed during dataset construction to constrain the structure, scope, and intent of generated answers. This approach serves as a form of embedded structural safety, guiding responses toward clinically appropriate, risk-aware, and ethically aligned outputs. By reducing contextual noise and limiting open-ended generation, the schema functions as a preventive mechanism against unsafe extrapolations and ambiguous medical advice—risks that remain salient even in expert-curated datasets.

However, despite both expert evaluation and constrained generation, it is important to acknowledge that large language models inherently retain probabilistic limitations that may affect output accuracy. Accordingly, the proposed dataset was not designed for autonomous or unsupervised clinical use. Any downstream clinical implementation should incorporate human-in-the-loop oversight, independent validation, and regulatory scrutiny to mitigate automation bias and ensure patient safety.

From an ethical standpoint, this layered approach—combining physician evaluation with schema enforcement—aligns with recently proposed “ethical firewall” frameworks for trustworthy AI in medicine and education. Thurzo [44] conceptualizes ethical firewalls as formalized boundary-setting mechanisms that constrain AI behavior without claiming infallibility. In this context, the constrained generation strategy adopted in the present work can be interpreted as an ethical and technical safeguard embedded at the data level, complementing expert judgment rather than replacing it.

By situating the proposed medical Q&A dataset within this dual framework of expert validation and ethical constraint, the study emphasizes a responsible pathway for developing trustworthy AI resources in healthcare. This integrated perspective highlights that clinical credibility, ethical governance, and human oversight must coexist to support the safe use of generative AI in medical education and decision-support settings.

Methodologically, the study introduced a novel two-stage “distill-then-generate” pipeline, which demonstrated clear advantages over direct generation methods widely used in biomedical NLP. In contrast to datasets such as MedExQA [29] and MedxpertQA [30], which rely on human-authored explanations to enhance interpretability, our approach incorporates an LLM-based distillation layer to support contextual refinement during dataset construction. Informed by prior work on long-context challenges, including the “lost in the middle” phenomenon [32], this pre-filtering step is designed to reduce irrelevant information and to emphasize clinically relevant content. This design choice aims to provide more focused contextual input for question generation, without implying direct empirical measurement of improvements in contextual coherence or factual precision. While extensive ablation studies on chunking parameters were beyond the scope of this resource paper, the selected parameters were empirically derived to optimize the trade-off between semantic completeness and the context window limitations identified.

Ultimately, the strategic value of the PMR-Q&A dataset lies in its potential to facilitate the training of Small Language Models (SLMs). By leveraging this high-fidelity, domain-specific corpus, future research aims to demonstrate that computationally efficient SLMs can achieve reasoning performance parity with larger foundation models within the rehabilitation domain, offering a scalable and specialized solution for clinical deployment.

Additionally, the integration of a schema-enforced generation protocol ensured structural consistency and type safety across all entries. Prior large-scale datasets such as MedMCQA [19] and emrQA [39] often suffered from formatting inconsistencies and semantic ambiguities. By implementing JSON Schema-based declarative constraints, our system eliminated post hoc parsing errors, producing machine-readable and uniformly structured Q&A pairs. This makes the PMR dataset not only clinically relevant but also immediately compatible with machine learning frameworks for downstream fine-tuning and benchmarking. Furthermore, future iterations of the pipeline could incorporate advanced text analytics and novel transformer architectures for automated safety filtering. For instance, Ref. [45] proposed a hybrid neural network transformer for detecting and classifying destructive content, a methodology that could be adapted to automate the detection and exclusion of non-clinical or erroneous outputs in generated medical datasets.

In terms of clinical representation, the dataset places substantial emphasis on scenario-based, application-oriented, and reasoning-intensive questions. This aligns conceptually with Suri et al. [46], who proposed the inclusion of realistic clinician–patient dialogues in the MeDiaQA dataset. However, in contrast to their dialogue-based approach, our study deliberately excluded conversational transcripts to preserve professional tone and medical precision. Quantitative reasoning questions were selectively included, reflecting real clinical computation tasks; nevertheless, expanding this subset represents a potential avenue for future iterations.

A further distinguishing aspect of the PMR dataset is its bilingual structure (Turkish–English). To date, most medical Q&A datasets have been developed exclusively in English [47], which limits the representation of non-English clinical contexts and hinders the development of localized AI systems. By offering parallel bilingual content, this study contributes to both the national development of Turkish-language clinical NLP resources and the global standardization of rehabilitation-oriented datasets.

In summary, this research presents a robust and clinically evaluated dataset that bridges the gap between general medical Q&A corpora and domain-specific reasoning requirements in rehabilitation medicine. The methodological rigor, structural uniformity, and expert-driven evaluation framework collectively position this dataset as a foundational resource for the training and benchmarking of large language models in PMR. Its integration into future AI systems has the potential to significantly enhance clinical education, diagnostic reasoning, and intelligent decision-support mechanisms within the field.

## 5. Conclusions

This study demonstrates that a clinically relevant, original Turkish–English Question–Answer dataset in the field of PMR can be systematically and automatically generated. The resulting dataset of 143,712 pairs is distinct from existing medical Q&A datasets in the literature, both in terms of its specificity to the FTR field and its inclusion of Turkish language support, as well as its incorporation of expert evaluation. The two-stage “distill-then-generate” approach applied in the study was designed to support semantic consistency by improving contextual focus, while the schema-constrained generation method helped promote structured and consistent data representation. Furthermore, the use of clinical scenario-based question structures adds depth to the dataset by supporting context-aware information representation, rather than simple fact retrieval.

Expert evaluations show that both the English and Turkish datasets have consistent quality and domain relevance according to expert assessments. Focusing on disease groups frequently encountered in the field of PMR increases the usability of the dataset in both educational and decision support systems. In this respect, the study proposes a new methodological paradigm not only in the field of PMR but also for training specialized artificial intelligence models in medical subdisciplines in general. The fact that it has been prepared in both Turkish and English enhances the study’s potential to contribute to both national and international artificial intelligence research.

### 5.1. Future Studies

Future studies are expected to further develop the scope of the dataset through multimodal (e.g., image or signal-based) data integration and large-scale expert validation processes. Nevertheless, this study serves as a pioneering and fundamental resource for AI-supported education, decision-making, and clinical guidance systems to be developed in the field of PMR.

### 5.2. Limitations

While this study introduces a rigorously developed and clinically evaluated dataset, certain limitations should be acknowledged. A primary limitation of this study is the absence of large-scale downstream benchmarking, such as fine-tuning performance or retrieval-augmented generation (RAG) accuracy evaluations. While the intrinsic quality of the data has been rigorously validated by domain experts, its extrinsic utility in training state-of-the-art models remains to be empirically quantified. Future studies will focus on establishing these baselines by benchmarking the PMR-Q&A dataset against existing medical corpora. The dataset currently focuses on text-based sources, and future iterations could benefit from the inclusion of multimodal clinical data to enhance contextual diversity. Although the “distill-then-generate” approach effectively minimized contextual noise and bias, inherent constraints of large language models may still influence output precision. The dataset is not intended for autonomous or unsupervised clinical use. Any downstream application in clinical settings should involve appropriate human oversight, independent validation, and regulatory review to mitigate automation bias. Expert evaluation, though conducted on a statistically representative subset, may be further strengthened through broader, multi-institutional collaboration. Finally, as the dataset is grounded in existing scientific literature, periodic updates will be essential to ensure alignment with evolving clinical evidence and rehabilitation practices.

## Figures and Tables

**Table 1:** Question–answer dataset studies created in the medical discipline.

Ref.	Purpose	Data	Large Language Model	Results
Pal, Umapathi and Sankarasubbu [19]	Introducing “MedMCQA,” a large-scale question-and-answer dataset covering real-world medical entrance exam questions, with multiple topics and multiple choices	Multiple-choice medical entrance exam questions covering 21 different medical fields—correct answers	BERT, SciBERT, PubmedBERT, BioBERT	The created dataset goes beyond the limitations of existing medical Q&A datasets and focuses on questions that require more complex medical reasoning and cover a wide range of topics
Taihua Shao [16]	A Transformer-based neural network architecture is proposed to solve the “answer selection” problem, which is a critical subtask in Q &A systems	WikiQA Dataset	QA-TFWP QA-TFMP QA-TFAP	The QA-TFAP model, which showed the best performance, achieved a 2.37% increase in MAP, a 2.83% increase in MRR, and a 1.51% increase in accuracy. In particular, it showed a clear superiority over traditional models, with an 11.08% increase in MAP, a 12.75% increase in MRR, and a 19.55% increase in accuracy, especially in long answers
Andrew Wen [20]	Adapting and evaluating a deep learning-based language model (BERT) to answer “why” questions on clinical texts	- emrQAwhy - SQuADwhy - i2b2notespre	BERT ve Clinical BERT	The best model achieved 70.7% accuracy in exact matches and 76.0% accuracy in partial matches. While Clinical BERT’s accuracy was 6%, pre-training with SQuADwhy resulted in a 3% increase. Error analysis revealed 18% false negatives; however, in 12% of cases, the model responses were found to be more accurate than the gold standard
Jin et al. [17]	To create a large-scale open-domain question–answer dataset (MedQA) for developing artificial intelligence systems for doctors and medical students	Multiple-choice questions from the USMLE and medical specialty exams in China, Japan, and Taiwan	BERT, BioBERT and RoBERT Finetuning	The MedQA dataset, which forms an important basis for the development of open-ended question–answer systems in the field of medicine, has been successfully created
Bohao Zhou et al. [21]	Developing the Chinese Anesthesiology Benchmark (CAB), a Chinese dataset in the field of anesthesiology, and analyzing the performance of various LLMs through this dataset	Anesthesiology board exams, textbooks, hospital records, and online consultation platforms	17 different LLM (GPT-3.5-Turbo and GPT-4; Qwen, ChatGLM, InternLM; HuatuoGPT-13B, Bianque, PULSE, BenTsao models)	Closed-source general models (GPT-4) demonstrate the highest performance in the areas of knowledge, application, and security. The performance of open-source models varies depending on the task, with some reaching competitive levels (e.g., Qwen-7B-Chat). While medical models are strong in specific areas, their general knowledge coverage is limited; GPT-4 stands out in terms of accuracy and professionalism, particularly in patient–doctor interactions and case analysis
Alonso et al. [13]	To develop an explanatory and multilingual evaluation framework for measuring the performance of artificial intelligence-based language models in medical decision support processes in languages such as Spanish, French, and Italian	Based on the Médico Interno Residente (MIR) exams and created using the Antidote CasiMedicos dataset	LLaMA2 Mistral PMC-LLaMA BioMistral MedRAG method	Models with access to gold-standard information achieve over 90% accuracy, with the Mistral model outperforming others. RAG-7 and RAG-32 provide limited gains in zero-shot tasks and remain below gold-standard performance, while non-English performance is on average 10 points lower. During fine-tuning, RAG methods are more effective than training models directly on the dataset
Goenaga et al. [15]	To enhance the transparency and interpretability of AI decision-making by automatically extracting explanatory arguments for correct answers in specialist medical exams	Medical Specialty Exams (MIR) in Spain	Fine-tuning for transformer-based language models	The model’s ability to understand and extract argument structures in medical texts has been validated
Dong et al. [14]	An add-on module called “Discuss-RAG” is proposed to improve the performance of medical QA RAG systems with agent-based reasoning and enable LLMs to answer medical questions more accurately and reliably	Medical question–answer datasets such as MedQA-USMLE, PubMedQA, and MedMCQA	OpenAI-GPT-4 Discuss-RAG	These are higher-performance metrics on standard medical QA datasets such as MedQA-USMLE, PubMedQA, and MedMCQA. The Discuss-RAG system has been shown to provide significant improvements in correct answer rates compared to traditional RAG approaches
Hang et al. [18]	A new dataset (BPQA) focusing on blood pressure readings has been developed	It contains specially created medical questions that include blood pressure readings. In addition to BPQA, MedQA-USMLE	BERT, BioBERT, MedAlpaca and GPT-3.5	In particular, it has been shown that GPT-3.5 demonstrates improved performance by utilizing context-specific tagging

**Table 2:** Expert Evaluation Scores and Explanations.

Score	Description	Explanation
0	Inconsistent/Unusable Level	The question or answer lacks logical coherence and contains major scientific, clinical, or terminological errors. The source reliability is low, rendering the content unsuitable for evaluation
1	Partially Consistent/Insufficient Level	The question or answer demonstrates general consistency; however, justification and conceptual depth are limited. Scientific accuracy is partially achieved, requiring further elaboration or source support
2	Consistent and Adequate Level	The question or answer is logically consistent and based on fundamental scientific knowledge. The content is clearly expressed and reliable, with sufficient scope but without advanced analytical depth
3	In-Depth and Expert Level	The question or answer exhibits high logical consistency and provides comprehensive, analytical, and expert-level reasoning. The content is scientifically robust, contextually sound, and directly relevant to clinical practice

**Table 3:** Examples of Appropriate and Inappropriate Question Types Following the Question Standardization and Transformative Q&A Filtering Process.

Question	Classification
What methodological limitations and strengths were identified in the study evaluating the effectiveness of the counterforce brace?	Inappropriate
How were blinding and concealment ensured in the study, and were any adverse events related to the treatment reported?	Inappropriate
What novel finding regarding the use of the Patient-Specific Functional Scale (PSFS) for assessing functional improvement was demonstrated in this study?	Inappropriate
Why is weight management particularly important in patients diagnosed with early-onset osteoarthritis before age 55, and how should this influence their treatment plan?	Appropriate
Painful arc between 60 and 120° of shoulder elevation is most commonly a clinical sign of which shoulder pathology, and which anatomical structures are involved in the underlying impingement?	Appropriate
What is the effect of ESWT combined with exercise on pain and grip strength in patients with lateral epicondylitis, and how does this compare to placebo with exercise?	Appropriate

**Table 4:** Q&A dataset pair and examples of other data.

Question	Answer	Category	Question Type
What are the long-term outcomes of meniscal repair in elite football players, and how should this influence return-to-sport planning?	Meniscal repair in elite athletes shows positive long-term outcomes, indicating that return-to-sport planning should emphasize long-term follow-up and rehabilitation	Meniscopathy	Prognosis
In a patient with an ACL rupture, what is the clinical significance of detecting a medial meniscal ramp lesion on MRI, and how should this influence treatment planning?	Medial meniscal ramp lesions are common in ACL rupture cases and significantly impair knee stability, especially anterior stability	Meniscal Injury	Diagnostic Methods
In middle-aged and older patients, where clinical tests for meniscal tears are unreliable, which imaging modality can provide valuable diagnostic support?	Magnetic resonance imaging (MRI) provides significant diagnostic value when clinical tests for meniscal tears have limited reliability	Meniscopathy	Diagnostic Methods
In a patient diagnosed with a PCL tibial bony avulsion injury, which surgical approach is preferred to identify and treat concomitant intra-articular pathologies, and why?	Arthroscopic fixation is preferred for PCL tibial bony avulsions because it enables better identification and management of associated intra-articular injuries	Cruciate Ligament Injuries	Diagnostic Methods
If microRNA therapy is considered to accelerate healing in a patient with a meniscal tear, which miRNA promotes type II collagen production and healing in the meniscal white–white zone?	miR-220 promotes type II collagen production and healing in the meniscal white–white zone	Meniscal Pathology	PT Treatment

**Table 5:** Per-condition expert evaluation scores for the Top-15 disease categories.

Disease Category	n	Mean	SD
Lumbar Disc Herniation	305	1,990	0,287
Knee Osteoarthritis	289	1,938	0,428
Lateral Epicondylitis	207	1,396	0,768
Cervical Disc Herniation	194	1,851	0,357
Myofascial Pain Syndrome	182	1,951	0,337
Fibromyalgia	154	2,039	0,194
Piriformis Syndrome	142	1,951	0,300
Shoulder impingement	141	1,858	0,389
Sacroiliac Dysfunction	139	2,007	0,330
Cross-Ligament Injuries	138	1,920	0,343
Gonarthrosis	137	1,964	0,391
Coxarthrosis	119	1,941	0,300
Knee osteoarthritis	91	1,989	0,459
Meniscal Pathology	86	1,907	0,330
Hand Osteoarthritis	80	1,963	0,434

**Table 6:** Q&A dataset studies containing expert evaluations.

Dataset	Sources	Number of Completed Q&A Pairs/Quantitative Information	Expert Evaluations Results/Quantitative Information
PubMedQA [25]	Titles and abstracts of PubMed articles	273,500 pairs	1200 pairs verified by medical students. 230 pairs were rated as “ideal.”
EmrKBQA [42]	Patient data from the MIMIC-III Knowledge Base (KB)	Over 940,000 questions, logical forms, and answer triplets	1000 pairs manually labeled by medical experts (Yes/No/Maybe labels)
MedExQA [29]	Five different medical specialties that are underrepresented in current datasets. (Biomedical Engineering, Clinical Laboratory Science, Clinical Psychology, Occupational Therapy, and Speech-Language Pathology.)	965 question-and-answer pairs	100 test samples were rated by humans (scoring: 0, 0.5, 1)
MedXpertQA [30]	Specialty board exam questions and existing benchmarks with rigorous filtering/augmentation	4460 questions (4144 Text + 316 Multimodal)	All 70 question templates have been approved by an ECG specialist to ensure clinical utility
RealMedQA [38]	NICE (National Institute for Health and Care Excellence) clinical guideline recommendations	Total 101,475 generated pairs (1150 Human + 100,325 LLM); 1200 pairs verified	1200 question–answer pairs (4 evaluators)

## Data Availability

All data analyzed in this study are included in this article. We uploaded the PMR-Q&A dataset and code content to the Hugging Face profile (See link: https://huggingface.co/datasets/serhanayberkkilic/physiotherapy-evidence-qa (accessed on 22 November 2025)).
